# Advancements in mRNA Encoded Antibodies for Passive Immunotherapy

**DOI:** 10.3390/vaccines9020108

**Published:** 2021-01-31

**Authors:** Cailin E. Deal, Andrea Carfi, Obadiah J. Plante

**Affiliations:** Moderna, Inc., 200 Technology Square, Cambridge, MA 02114, USA; Cailin.Deal@modernatx.com

**Keywords:** mRNA, antibody, immunotherapy

## Abstract

Monoclonal antibodies are the fastest growing therapeutic class in medicine today. They hold great promise for a myriad of indications, including cancer, allergy, autoimmune and infectious diseases. However, the wide accessibility of these therapeutics is hindered by manufacturing and purification challenges that result in high costs and long lead times. Efforts are being made to find alternative ways to produce and deliver antibodies in more expedient and cost-effective platforms. The field of mRNA has made significant progress in the last ten years and has emerged as a highly attractive means of encoding and producing any protein of interest in vivo. Through the natural role of mRNA as a transient carrier of genetic information for translation into proteins, in vivo expression of mRNA-encoded antibodies offer many advantages over recombinantly produced antibodies. In this review, we examine both preclinical and clinical studies that demonstrate the feasibility of mRNA-encoded antibodies and discuss the remaining challenges ahead.

## 1. Introduction

Antibodies are components of the human adaptive immune system that are crucial to prevent, control and resolve infections [1]. Prior to the introduction of antibiotics and vaccines, passive transfer of serum containing antibodies from convalescent individuals or animals was the “standard of care” against infectious diseases [2]; this approach is still used today to treat venomous snake bites, toxin exposure, rabies and, more recently, the Ebola virus and SARS-CoV-2 infections [3,4,5]. Over the last two decades, the field of antibody-mediated immunotherapy has been transformed by the development of methods to immortalize B cells [6]. Further breakthroughs in recombinant antibody technologies, such as antibody isolation and gene sequencing, have resulted in the regulatory approval and commercialization of over 100 monoclonal antibodies (mAbs) to treat autoimmune diseases, neurodegenerative disorders, cancer and infectious disease [7,8,9,10]. To date, all licensed mAbs are purified IgG proteins that are administered intravenously (IV), intramuscularly (IM) or subcutaneously (SC) [2,8]. 

**Figure 1:**
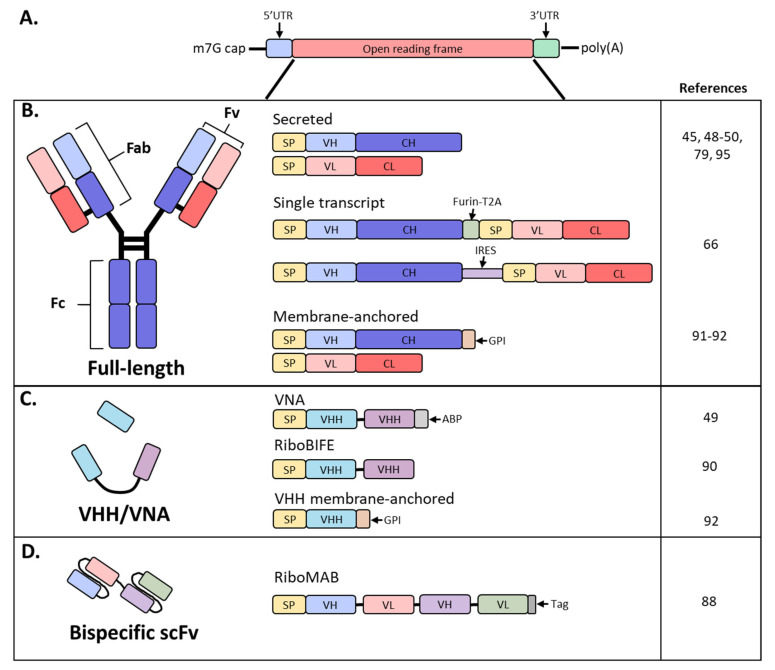
Schematic diagram of mRNA and antibody designs used in recent studies. (**A**) Basic structure of an mRNA construct; (**B**) different formats used by recent studies to encode a full-length antibody as mRNA; (**C**) derivatives of full-sized antibodies that are comprised of only the heavy chain; (**D**) bispecific antibody, single-chain variable fragment (scFv) format; SP, signal peptide; VH, variable heavy chain domain; CH, constant heavy chain domain; VL, variable light chain domain; CL, constant light chain domain; Furin-T2A, furin and thosea asigna virus 2A peptide; IRES, internal ribosomal entry site; GPI, glycosylphosphatidylinositol membrane anchor; VHH, VH domain, heavy chain only.

Antibodies are hetero-tetrameric proteins formed by two full-length heavy (H) and light (L) chains held together via charge–charge interactions and disulfide bonds. Two distinct parts of an antibody are critical for its function: the antigen binding fragment (Fab) and the crystallizable fragment (Fc) (Figure 1). The Fab region determines antibody specificity and is composed of one constant and one variable domain (Fv) of the H and L chains. The Fc region, comprised of the constant domains of two H chains, determines the in vivo antibody half-life by binding to the neonatal Fc receptor (FcRn) [11,12] and can modulate immune cell activity through binding to Fc receptors on innate immune cells [13]. The Fc effector functions can be altered by post-translational modifications such as glycosylation, methionine oxidation or deamidation, which can also impact antibody distribution and stability [14,15,16].

mAbs are routinely produced in Chinese hamster ovary (CHO) and other mammalian cells due to their high expression levels in stable cell lines, robust cell growth amenable to large-scale production and post-translational modifications akin to humans [17]. However, manufacturing of recombinant antibodies requires large volumes, costly production and complex protein characterization. Short antibody fragments, such as single-chain variable fragments (scFv’s) consisting of only the H and L chain variable regions or H chain only variable domain (V_H_H) derived from camelids or sharks, can be produced in *E. coli* or insect cells, both representing a cheaper approach for production (Figure 1B,C) [18]. These formats offer a similar antigen-binding affinity as that of the parent IgG, with the advantage of better tissue penetration due to their small size. Unfortunately, these antibody fragments suffer from short plasma half-lives due to the lack of FcRn-mediated recycling and necessitate frequent administration to maintain therapeutically relevant plasma levels [18].

Overall, antibodies have revolutionized drug discovery and development by enabling highly specific treatments for cancer and autoimmune disease while being continuously redesigned and engineered for enhanced affinity, stability and expression, making them the fastest growing class of therapeutics. However, there are still areas in need of improvement for recombinant antibodies, such as aggregation during long term storage, a broader biodistribution and the current difficulties in manufacturing antibody combination formulations [19,20,21]. Additionally, despite the improvements in mAb production, the need for repeated administration keeps the cost of this type of treatment relatively high [21].

An alternative to producing and purifying recombinant proteins for passive transfer is to use gene-based approaches. Delivering the genetic sequence of the antibody into an individual, using a viral vector, DNA or RNA, circumvents some of the challenges associated with large-scale production and characterization of traditional recombinant approaches and potentially allows for the design and generation of more complex antibody molecules that may exhibit improved efficacy [22]. In addition, these approaches may result in faster antibody development and are well-suited to respond to pandemic situations.

Viral vectors such as adenovirus (Ad) and adeno-associated virus (AAV) have been engineered for in vivo mAb expression by replacing part of their genome with the genes of antibodies of interest (Table 1) [23]. Both approaches have shown impressive levels of expression in the microgram per mL range in preclinical animal models [24,25,26,27,28]. However, human clinical trials utilizing Ad and AAV to express mAbs have led to variable and low in vivo expression levels, calling into question the validity of these approaches [26,27,28]. Unfortunately, both viruses are ubiquitous among the human population [29,30] and pre-existing immunity to the vector likely results in low virus uptake and ultimately limits mAb expression [31,32,33,34]. In addition, the immunogenicity of the viral vector, along with other safety concerns such as the risk of insertional mutagenesis for AAV [35,36], limits this approach for mAb immune therapies.

Gene transfer of the antibody nucleotide sequences themselves, as DNA or RNA, represents an alternative option for transient in vivo antibody production (Table 1). Both plasmid DNA and mRNA offer ease of large-scale production free from viral contamination in cell culture. Most plasmid DNA approaches utilize an electroporation device to enhance transfection efficiency following IM delivery [37,38]. To further increase transfection efficiency, hyaluronidase, an enzyme that breaks down an essential component of the extracellular matrix to enhance the distribution of plasmid DNA, is often used [39]. While IM electroporation of plasmid DNA results in physiologically relevant antibody titers in mice, it has been difficult to achieve similar levels in larger animals and humans [40]. Furthermore, there are safety risks to take into consideration, as DNA plasmid transfections, especially via electroporation, can increase integration of plasmid DNA into the genome, enhancing the risk of insertional mutagenesis [41].

Recently, mRNA delivery has become an attractive means of in vivo protein expression (Table 1). mRNA is a transient carrier of genetic information for the synthesis of proteins and does not require nuclear phase activity for efficient and rapid expression in nondividing cells. Additionally, there is no risk of insertion into the human genome or anti-vector immunity against the plasmid or viral backbone as seen with viral vectors or DNA plasmids. While initially considered too unstable due to rapid degradation by ubiquitous ribonucleases, modifications to the mRNA synthesis and purification processes, optimization of the mRNA codon usage and advancements in mRNA delivery methods, such as the use of lipid nanoparticles (LNPs), have led to more stable mRNA molecules and increased protein expression [42,43,44]. These improvements, together with efficient delivery without the need of a device, have enormously simplified and expanded the use of mRNA for therapeutic applications including antibodies.

Important differentiations between mRNA and other approaches are the kinetics, level and duration of protein expression. In vivo mAb expression from mRNA can be detected as early as 2 h post-administration since mRNA does not require nuclear localization for translation [45]. In contrast, mAb expression from DNA plasmids, Ad and AAV is detectable three, one and seven days post-inoculation, respectively [24,25,46]. Finally, the efficiency achieved with mRNA delivery enables greater protein production during peak expression as compared to naked plasmid DNA [47]. Of note, unlike recombinant protein injections, which result in almost immediate high-circulating mAb titers, mRNA-encoded antibodies result in peak expression around 24 to 48 h post-administration and expression from mRNA can last for several hours or days [45,48,49,50].

Despite the progress with mRNA technology there are still opportunities for improvements. Currently, administration of mRNA-encoded antibodies has been mostly limited to liver-targeting via the IV route with infusions of an hour or more, constraining the number of subjects that can receive treatment and diseases that can be treated. Also, higher levels of in vivo expression are needed to compete with recombinant antibodies and enable antibody combinations. Nevertheless, improvements in mRNA delivery and design are rapidly expanding the application of mRNA-based therapies and it is likely that the remaining challenges will be overcome in the near future [45,51,52].

## 2. mRNA as a Platform for Efficient Protein Expression In Vivo

mRNA synthesis starts with in vitro transcription (IVT) by RNA polymerases of a linear DNA template that contains a promoter, 5’ and 3′ untranslated regions (UTRs), and an open reading frame [53]. Two additional elements are required for the mRNA to be biologically active: an inverted triphosphate cap at the 5′ end and a poly(A) tail at the 3′ end. Both a cap and poly(A) tail are required for efficient translation and stabilization of the mRNA, and both elements can be added either during transcription or enzymatically after [54]. Importantly, cap structure [55] and poly(A) tail lengths [56] can impact the amount of protein produced by an mRNA-based therapy (Figure 1A). Other factors that can affect mRNA translation or half-life include UTRs [57,58,59] and codon optimization [60]; the work of understanding and balancing these mRNA elements is an area of intense research.

Protein expression from exogenous mRNA was demonstrated in vivo two decades ago when direct injection of mRNA into mouse muscle was shown to result in local protein expression [61]; however, there are still several hurdles that need to be solved. First and foremost, mRNA is an unstable molecule that is degradable by ribonucleases in the body resulting in a very short half-life. Additionally, naked mRNA is not efficiently delivered to cells and mRNA can activate innate immune pathways that may decrease protein expression [52]. In the last decade, significant progress has been made in all these areas.

## 3. Modified mRNA

mRNA and the side products of the mRNA in vitro transcription (IVT) process can trigger an innate immune response through pathogen-associated molecular pattern (PAMP) receptors, such as toll-like receptor (TLR)3, TLR7, TLR8, retinoic acid-inducible gene 1 (RIG-I) and nucleotide-binding and oligomerization domain containing protein 2-(NOD-2), which recognize either double-or single-stranded RNA [52]. Activation of the innate immune pathways could interfere with therapeutic applications by decreasing protein expression and undermining tolerability. Modified bases found in natural RNAs have been revealed to not only suppress recognition by TLRs in vivo but also to increase the stability and translation of mRNA [42,43]. A common modification is replacement of uridine with pseudouridine, which has been shown to increase mRNA translation and decrease innate immune stimulation [43,62]. Further reduction in stimulation of innate immunity has been obtained by stringent mRNA purification by high-performance liquid chromatography (HPLC), which can remove the aberrant RNAs created in the IVT reaction [63,64].

## 4. Self-Amplifying mRNA

**Figure 2:**
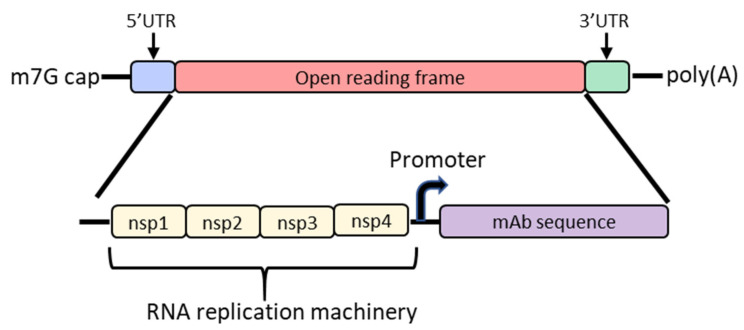
Diagram of the basic structure of self-amplifying mRNA (RepRNA) that includes the typical cap, 5′ and 3′ UTRs and poly(A) tail. The details of the open reading frame are depicted and contain the nsp1, nsp2, nsp3 and nsp4 genes from the alphavirus genome that encode for RNA replication machinery. Downstream from the rep genes are a subgenomic promoter and the elements encoding a monoclonal antibody (mAb). Abbreviations: m7G: 7-methylguanosine; UTR: untranslated region; nsp: nonstructural protein.

In order to increase protein expression and decrease mRNA dose, some groups have developed a self-amplifying mRNA (SAM) approach based on the alphavirus genome that encodes its own RNA replication machinery [65,66]. Due to the bipartite division of structural and nonstructural regions in the alphavirus genome, structural genes can be replaced with genes of interest while still retaining the machinery for replicative functions (Figure 2). However, there are significant drawbacks to this approach, largely due to the sheer size of the construct and their intrinsically high innate immune stimulation. To encode the polymerase, replicons start at a size of ~7.6 kb; the addition of the gene of interest results in a large construct that is prone to cleavage and therefore unstable [67,68,69]. As such, large-scale manufacturing, storage and characterization of these constructs are quite complex and expensive and constitute an active area of research in the field [65,68]. To overcome the difficulties of SAM resulting from size, the replicase can be provided in trans [70]. While this results in shorter mRNAs, it requires manufacturing of at least two RNAs and efficient delivery of both into the same cell. Another disadvantage of SAMs is their intrinsic immunogenicity due to the formation of short double-stranded RNA during production and self-amplification [68]. There will need to be significant improvements in the stability of long mRNAs, immunogenicity and more complex manufacturing before SAM can become a realistic option in the clinic to produce monoclonal antibodies in vivo.

## 5. mRNA Delivery with LNPs

A large size and negative charge of mRNA are obstacles to it efficiently reaching the cytosol [53]. Naked mRNA can be spontaneously taken up by different cell types but this usually results in degradation in acidic endolysosomal compartments [71,72]. Lipid nanoparticles (LNPs) have been identified as an efficient way to protect mRNA from ubiquitous RNAses, shield it from immune cells and deliver it to cells while enabling escape from the endosome [45]. LNPs generally consist of four major components: an ionizable, a sterol, a phospholipid and a lipid-anchored polyethylene glycol (PEG) [73]. The phospholipid and sterol work together to stabilize the LNP, the lipid-anchored PEG provides vial and storage stability and the ionizable lipid is critical for cellular uptake and endosomal escape, allowing for release of the mRNA into the cytosol [73,74]. By changing the ratio and identities of the lipid components, the efficacy and tolerability of the formulation can be altered [73,74]. Importantly, mRNA/LNP formulation requires careful process controls to ensure reproducibility of manufacturing and stability [75].

## 6. mRNA/LNP Mediated In Vivo Antibody Expression

The last ten years has resulted in a flurry of mRNA research for therapeutic and vaccine applications [76]. However, only a few pre-clinical studies have been reported for mRNA-encoded antibodies and only one program has moved into a phase I clinical trial (NCT03829384; Table 2; Figure 1).

## 7. Full Length Antibodies

There are multiple strategies that have been used to express mRNA-encoded antibodies in vivo. The Weissman group was the first to publish on an mRNA-encoded antibody approach [48]. They co-delivered 30 µg of a 1:1 molar ratio of mRNA encoding for the H and L chain of an HIV bNAb VRC01 IV to BALB/c mice and achieved peak plasma antibody titers greater than 150 µg/mL at 24 h. Antibody levels were stable for 5 days before a precipitous decrease that resulted in clearance by day 11. This was also observed in immune-deficient mice indicating that clearance was not a result of anti-drug antibody (ADA) responses. Humanized mice that express human lymphoid cells were administered 7.5, 15 or 30 µg of mRNA-encoded VRC01 which led to a dose-dependent increase in antibody expression in vivo. Interestingly, 30 µg of mRNA-VRC01 led to almost twofold higher concentration of circulating VRC01 when compared to injecting 600 µg of recombinant protein. These mice were all protected from HIV challenge except for those who received the 7.5 µg dose (expressing 23.5 µg/mL). Protection was confirmed in a second humanized mouse model with a different strain of HIV [48].

Thran et al. evaluated three mRNA-encoded mAbs: S057 (anti-rabies), CR8033 (anti-influenza A) and rituximab (anti-human CD20) [49,77]. Interestingly, all mRNA used in this study did not contain modified nucleotides. A systematic in vitro titration of mRNA encoding for the H and L chains revealed that a 1.5:1 molar ratio was optimal for antibody expression. Dose escalation from 1.25 µg (0.0625 mg/kg) to 40 µg (2 mg/kg) resulted in a dose-dependent increase in serum concentrations, achieving 10 µg/mL in the 2 mg/kg group. Importantly, no toxicity was found related to the mRNA infusion; an LNP was utilized that was specifically developed for liver transfection and histopathology of the liver did not reveal any abnormality. Notably, S057 at 2 mg/kg protected mice from rabies infection in pre- and post-exposure prophylaxis models [49].

To investigate the potential of the mRNA-encoded antibody approach for cancer indications, rituximab, an antibody therapeutic used to treat B cell lymphomas [77], was used in a non-Hodgkin’s lymphoma xenograft mouse model [49]. Control over tumor growth was superior with 2.5 mg/kg of mRNA given twice a week as compared to 10 mg/kg of recombinant protein administered on the same schedule. Due to the slight delay in tumor growth kinetics resulting from an irrelevant antibody mRNA, the authors concluded that a weak cytokine response may have impacted the tumor growth but, nevertheless, could not explain the superiority of mRNA treatment as compared to traditional protein [49].

The application of mRNA-encoded antibodies in cancer was further explored with trastuzumab, an anti-HER2 antibody currently used to treat breast cancer patients [78,79]. Mice were IV injected with formulated mRNA at a pre-determined 2:1 molar ratio of mRNA for the H and L chains, respectively. A dose-dependent increase in expression was observed at all doses tested, with a 2 mg/kg dose reaching almost 60 µg/mL at peak. When compared to 8 mg/kg of trastuzumab protein (Herceptin), the mRNA-encoded antibody exhibited a 64% higher serum concentration over the course of 30 days, demonstrating more favorable pharmacokinetics than the recombinant antibody. mRNA-expressed antibody retained its potent antibody-dependent cell cytotoxicity (ADCC) properties in vitro and led to a statistically significant delay in HER2-positive tumor cell growth in vivo when administered weekly [79].

Preclinical studies often show favorable characteristics in rodents but these results often do not translate to larger species. Kose et al. investigated the utility of the mRNA-encoded mAbs approach with CHKV-24, a neutralizing mAb against the Chikungunya virus [50]. A dose escalation study in mice demonstrated ranges of expression from less than 1 µg/mL with a 0.02 mg/kg dose to almost 15 µg/mL with a 0.5 mg/kg dose, of which the highest mRNA dose provided protection from viral challenge. Partial protection was achieved at the 0.1 mg/kg dose and while there was no protection at the lowest dose, there was a statistically significant delay in death. Furthermore, the authors demonstrated therapeutic efficacy against polyarthritis, the most common manifestation of Chikungunya virus infection. Importantly, a single IV infusion of 0.5 mg/kg CHKV-24 mRNA in NHPs resulted in peak concentrations of ~40 µg/mL of antibody in circulation with a calculated half-life of 23 days [50].

A potential risk with repeated LNP dosing is their ability to activate the complement system which could elicit a hypersensitivity reaction known as complement activation-related pseudo-allergy (CARPA) [80,81]. Administration of CHKV-24 mRNA at 0.3 mg/kg, 1.0 mg/kg or 3.0 mg/kg in NHPs resulted in an additive, dose-dependent increase in CHKV-24 serum concentrations. This was further increased when a second dose of mRNA at 3.0 mg/kg was administered, resulting in peak average levels of 28.8 µg/mL [50]. No significant adverse events were observed when the two infusions were separated by one week. The safety profile and the capacity to dose repeatedly allow for mRNA-encoded mAb technology to be applied to other indications requiring either high circulating levels or multiple doses, such as cancer or autoimmune diseases.

SAM approaches have been also exploited for the expression of full-length antibodies. The ZIKV-117 mAb sequence was inserted downstream from the replicon machinery of a vaccine strain of Venezuelan equine encephalitis virus and was termed RepRNA [66]. ZIKV-117 is a potent and broadly neutralizing mAb against the Zika virus that has previously been shown to protect against lethal viral challenges in mouse models [82]. Three different strategies were employed to express full-length antibodies: separate replicons encoding for the H and L chains or a single mRNA with H and L chains separated by either an internal ribosomal entry site (IRES) or a furin cleavage site and T2A viral peptide sequence to promote ribosomal skipping (Figure 1B,C) [83,84]. Supernatant from transfected BHK cells exhibited neutralizing activity for all constructs tested except for the separate replicons. The IRES and T2A coding strategies were further optimized and tested in vitro and in vivo by screening IRES sequences, signal peptides and the orientation of H and L chain sequences around the IRES or T2A. Dose escalation studies demonstrated increasing serum concentrations of ZIKV-117 as the dose escalated to 10 µg of mRNA, after which there was no significant increase, likely due to saturation of protein production from cells at the injection site and innate immune activation [85]. All subsequent studies employed four separate IM injections of 10 µg each for a total of 40 µg (2 mg/kg) per mouse [66].

Expression levels from an IM injection of RepRNA containing the T2A sequence were compared with traditional modified mRNA. In this study, RepRNA achieved up to 32-fold higher serum concentrations of mAb over mRNA. RepRNA containing pseudouridine modifications resulted in a lack of detectable antibody in mouse serum likely due to differential tertiary folding of the IRES caused by the additional hydrogen bond donor of N1 in pseudouridine compared to uridine. Lastly, the IRES RepRNA construct demonstrated robust protection when given as a prophylaxis or therapy [66]. Despite the potential of SAM approaches, challenges in manufacturing, characterization and stability of these large mRNA constructs are serious hurdles to be overcome for this approach to be clinically feasible [68].

## 8. Single-Chain Antibodies

Although full-length antibodies have engendered significant success targeting cancers, immune disorders and infectious diseases, they are relatively large and exhibit less tissue penetrance than small molecules. Contrastingly, V_H_H and other single-domain antibodies have a small size that enables better tissue penetration [18]. These are the smallest antigen-binding fragments that retain affinities and antigen-binding specificities comparable to full-length antibodies. However, the use of these molecules has been hampered by their short half-lives of several hours and propensity to aggregate [18]. Both properties could be improved or avoided through mRNA-mediated expression since antibody will be continuously expressed from the mRNA until the mRNA is degraded; this approach would also prevent the aggregation often observed during protein purification (Figure 1C).

While V_H_H’s are traditionally single domain, Thran et al. fused two V_H_H’s to enable neutralization of toxins (VNA; Figure 1C) [49]. Additionally, an albumin-binding peptide was added to the 3′ end to increase serum antibody half-life due to the lack of an Fc. Functionality of VNAs against botulinum neurotoxin A (VNA-BoNTA [86]) and Shiga toxin 2 from O157:H7 *E. coli* (VNA-Stx2 [87]) were confirmed in vitro. Following a single IV administration of 40 µg (2 mg/kg), VNA-Stx2 exhibited similar kinetics and peak expression as the full-length constructs, reaching roughly 40 µg/mL by 24 h. Interestingly, VNA-BoNTA exhibited roughly 20-fold higher serum levels with a peak at 24 h between 200 and 400 µg/mL as compared to full-length antibodies. While kinetics of VNAs were similar to full-length mAbs, serum titers decreased rapidly due to the lack of FcRn recycling and exhibited a calculated half-life of 24–36 h. Botulism toxin is the most potent toxin known and induces rapid onset of symptoms, necessitating immediate treatment. To determine whether mRNA-encoded VNA-BoNTA would be amenable as an anti-toxin therapy, challenge studies were initiated whereby mice received a 2 mg/kg dose of mRNA VNA-BoNTA at 2, 4 or 6 h post-challenge. Due to the relatively rapid onset of VNA expression from mRNA, all mice survived but the mice treated 6 h post-challenge developed mild clinical symptoms; this was comparable to a lower dose (0.1 mg/kg) of VNA-BoNTA recombinant protein. While mRNA expression kinetics achieve Cmax with a delay compared to infused protein, mRNA may still be rapid enough to treat most post-exposure prophylaxis indications [49].

Recently, Stadler et al. investigated the use of mRNA-produced single-chain bispecific antibodies to target tumors in mice (Figure 1D) [88]. Importantly, the mRNA was purified and contained modified nucleosides; unpurified and unmodified mRNA expressed poorly and induced significant levels of cytokines. A tandem bi-(scFv)_2_ was designed, coined RiboMAB, in which two scFv’s with different antigen specificities were fused into a single construct (Figure 1C). Three different RiboMABs were generated, all with one arm targeting CD3, a T-cell receptor-associated molecule, to engage T cells, and the other arm binding to one of three tumor-associated antigens (TAA): tight-junction proteins claudin 6 (CLDN6), claudin 18.2 (CLDN18.2) or epithelial cell adhesion molecule (EpCAM). RiboMAB potency from in vitro transfected K562 cells was comparable to recombinant protein and induced dose-dependent, target-specific T-cell activation and lysis. In mice, IV administration of 0.25 mg/kg RiboMAB led to a peak concentration of ~7 µg/mL at 6 hpi and persisted in the serum up to six days post-inoculation. This is compared to recombinant protein which was almost undetectable by 24 h post-injection. To further investigate the therapeutic effect of this approach, immunodeficient mice were engrafted with human peripheral blood mononuclear cells and implanted SC with human OV-90 ovarian carcinoma cells expressing a specific TAA. Complete tumor elimination was achieved by one infusion a week of 0.15 mg/kg RiboMAB mRNA for three weeks. A comparable anti-tumor response with recombinant protein required three infusions a week of 0.2 mg/kg for a total of ten injections. More sustained levels of RiboMAB were achieved with mRNA than recombinant protein, likely due to the continuous production of antibody from mRNA until mRNA decay as compared to a single infusion of protein. Tumors collected from RiboMAB-treated mice exhibited a large T-cell infiltration indicating effective concentrations of RiboMAB within the tumor itself [88]. Thus, the inherent pharmacokinetic advantage of mRNA expression makes this technology well suited for the expression of small, short-lived antibodies.

## 9. Engineering mRNA Antibody for Local Delivery

Targeted delivery of therapeutics to organs of interest has the potential to minimize systemic toxicity and ADAs and reduce the amount of drug needed to reach therapeutic levels at relevant sites; this is especially applicable for prevention or treatment of mucosal pathogens. Of these, the lung and female reproductive tract (FRT) are potential targets for certain infectious diseases including influenza A, respiratory syncytial virus (RSV) and HIV. Van Hoecke et al. encoded an engineered bispecific V_H_H construct (RiboBiFE) that had previously been shown to recruit innate immune cells to influenza-infected cells by linking an influenza A matrix protein 2 ectodomain (M2e)-specific V_H_H to a V_H_H that selectively binds mouse Fcƴ receptor IV (FcƴIV; Figure 1C) [89,90]. Intratracheal (IT) injection of the recombinant bis-specific protein was barely detectable in the lung at 24 h post-injection in comparison to mRNA-encoded RiboBiFE that was detected in bronchiolar lavage fluid for 48 h, demonstrating the prolonged availability of the mRNA-encoded RiboBIFE in the lung compartment. Importantly, mice receiving FcƴIV-M2e RiboBIFE exhibited less weight loss, higher survival and a significantly lower viral load in the lung upon influenza A challenge [90].

RiboBIFE constructs did not persist in the lung past 48 h post-inoculation and many indications require longer antibody persistence. To retain antibody at mucosal sites, a glycosylphosphatidylinositol (GPI) membrane anchor sequence from decay-accelerating factor (DAF) was added to the H chain for the mRNA-encoded mAb (Figure 1B) [91,92]. Transfected membrane-anchored palivizumab (aPali), an FDA-approved mAb for prevention of RSV in high-risk infants, localized to the cell surface, demonstrating the functionality of the membrane anchor [92,93]. Approximately 45% of total isolated lung cells expressed aPali in two out of three mice following IT administration. This was in comparison to standard IM administration of labeled recombinant palivizumab in which low levels of antibody were found in the lung and the majority was localized to the injection site. Non-natural GPI membrane anchors were especially important to retain V_H_H antibody formats as single-chain antibodies suffer from short half-lives, ranging from 30 min to 2 h in serum (Figure 1C). In this study, membrane-bound V_H_H was still detectable in the lungs of mice 28 days after IT inoculation and was able to significantly reduce RSV titers when challenged seven days after mRNA administration [92].

Antibodies to prevent sexually transmitted infections could benefit from local administration and membrane-anchored retention. Predictive models have estimated that serum antibody is roughly 90-fold higher than in vaginal secretions [94], necessitating relatively high doses of systemic antibody. PGT121, an HIV bNAb, was aerosolized in water as an mRNA-encoded secreted (sPGT121) or membrane-bound (aPGT121) antibody and delivered to the cervix of sheep, which have a similar FRT structure as humans [91]. Pharmacokinetics of two 750 µg doses of aPGT121 and sPGT121 revealed that the GPI anchor retained aPGT121 at high concentrations in genital secretions for longer than sPGT121, with concentrations peaking at 24 h around 100 µg/mL and decreasing to 40 µg/mL by day 28. In contrast, sPGT121 decreased rapidly to ~10 µg/mL by day 14. To test the protective efficacy of this approach, female rhesus macaques were treated with 250 µg (0.05 mg/kg), 400 µg (0.075 mg/kg) or 1000 µg (0.2 mg/kg) of aPGT121 mRNA and FRT biopsies at 24 h post-administration were challenged with simian immunodeficiency virus expressing a clade B HIV envelope (SHIV). There was a dose-dependent increase in SHIV infection: the 250 µg explants were the most susceptible and the 1000 µg explants were refractory to infection [91]. This study further highlights the importance of membrane anchors for prolonged expression at mucosal sites and reveals a new modality to achieve relevant levels of antibody at the sites of infection for many pathogens.

## 10. mRNA-Encoded Antibodies in Clinical Trials

Positive interim clinical data was presented this year on the first ever clinical trial of an mRNA-encoded antibody [95]. mRNA-1944 encodes a potent Chikungunya virus antibody and contains the LS half-life extension mutation [50,96]. This phase 1 clinical trial (NCT03829384) was a randomized, placebo-controlled, dose-escalation study in healthy adults. All individuals received premedication with antihistamines prior to IV administration of the drug product. mRNA-1944 was generally well-tolerated with only mild adverse events (AEs) being reported. There were no significant changes in liver or kidney lab results across all groups tested. Titers peaked around 24 h at concentrations of 2, 7.9 and 10.2 µg/mL in groups receiving 0.1, 0.3 and 0.6 mg/kg, respectively. Preclinical data predicted that concentrations greater than 1 µg/mL of circulating antibody would be protective in humans; this target level was exceeded for at least 16 weeks following a single injection at 0.3 mg/kg. Of the dose regimens, 0.6 mg/kg resulted in the highest number of AEs. In the same study, a group of volunteers received two infusions of 0.3 mg/kg separated by one week. There was no significant accumulation of lipid components, induction of CARPA or serious AEs reported after the second administration. Akin to NHPs, there was additive expression when administering two doses, with the average peak titer reaching 7.2 and 12.9 µg/mL after the first and second doses, respectively [50,95]. The results for this human clinical trial of an mRNA-encoded antibody are very promising as they demonstrate, for the first time, the safety of this approach. A rapid increase in antibody titers following IV mRNA/LNP administration and biologically relevant titers were obtained with an antibody half-life of over 2 months.

## 11. Future of mRNA-Encoded Antibodies

While there have been relatively few mRNA-based antibody studies published, it is a rapidly growing field with significant promise. Work on mRNA-encoded protein expression and demonstration of its efficacy in mice and NHPs as well as expression and safety in humans has paved the way for mRNA-encoded antibodies. mRNA delivery with LNPs and the discovery of new and better-tolerated lipids have enabled a substantial increase in antibody expression in NHPs [45]. Similarly, advances in modified nucleotide chemistry have reduced innate immune stimulation and increased in vivo protein production, enabling mRNA to be pursued for therapeutic applications. All these advances together with progress in mRNA sequence designs, manufacturing and purification have culminated in the completion of the first clinical trial of an mRNA-encoded antibody.

In vivo studies have demonstrated that mRNA-encoded antibodies offer some benefits compared to other approaches. Unlike recombinant protein, which provides a single protein bolus upon administration, expression from mRNA results in expression for several days, dependent upon the half-life of the mRNA, after which normal antibody kinetics preside. The pharmacokinetics of mRNA could be highly beneficial when using short-lived antibody fragments such as scFv and VNA [49,88]. This could allow less frequent treatments when repeated dosing is required. Additionally, mRNA sequences are all comprised of the same nucleotides, resulting in sequences that have much more similar physiochemical characteristics than the proteins they encode for. As such, mRNA manufacturing is a more standard process that permits rapid production, making this technology well-suited for rapid response to pandemic situations where antibodies would be useful. This potential has been exemplified with mRNA antigens during the current SARS-CoV-2 pandemic in which an mRNA vaccine went from sequencing to first clinical trial in humans within 63 days and emergency use authorization (EAU) in less than a year [97]. Additionally, mixing of mRNA sequences could enable ease of manufacturing for antibody cocktails. However, technology needs to be developed to avoid unwanted chimeras upon administration of more than one co-formulated mRNA-encoded full-length antibody.

The LNP formulations that encapsulate mRNA for delivery can also offer advantages for this technology over recombinant proteins as they may offer greater biodistribution, with more tissue penetration than recombinant antibody proteins. However, higher expression with more convenient routes of administration, such as subcutaneous or IM, needs to be achieved to allow more widespread use of mRNA as an alternative to recombinant antibodies. Despite further optimizations that need to occur to allow mRNA-encoded antibodies to be a competitive therapeutic product, significant advances have been made and this is only the beginning for this emerging and potentially disruptive field.

## Figures and Tables

**Table 1:** Advantages and disadvantages of different antibody expression platforms.

Delivery Method	Advantages	Disadvantages
Recombinant protein	Immediate peak circulating antibody Repeated dosing SC and IM delivery possible High mass titers rapidly achievable	Duration of circulating level dependent upon serum half-life of antibody Length of production time and manufacturing
Viral vectored	High expression (>100 µg/mL) IM injection (AAV) Expression lasts for years (AAV) Well-tolerated clinical safety profile (AAV)	One week (Ad) or one month (AAV) to achieve peak expression Immunogenic vector results in one dose per serotype Can induce immune response to the antibody Vector pre-existing immunity inhibiting transduction Potential safety issues with long-term expression Risk of insertional mutagenesis (AAV) Size limitation of transgene (AAV)
DNA	Expression can last up to a year Potential for repeated dosing Easy to scale up production IM injection Cell-free production Same technology for all antibody formats—can encode alternative isotypes	Generally low expression (1–20 µg/mL) with 50–300 µg of DNA Requires electroporation device and hyaluronidase for increased uptake Takes ~7–14 days for peak expression Risk of insertional mutagenesis Risk of inducing autoimmune antibodies against DNA from repeated injection
mRNA	Expression within hours Repeated dosing Easy to scale up production Well-tolerated clinical safety profile Potentially different biodistribution Cell-free production	Short-lived expression IV administration No chemical modifications or antibody conjugates

**Table 2:** Overview of the current literature on mRNA-encoded antibodies.

	Antibody	Antibody Format (H:L Molar Ratio)	Antigen Target	Specific Modifications	Formulation	Species	Maximum Titer (Dose)	Citation
**Coexpressed**	VRC01	Full-length (1:1)	HIV (CD4bs)	N1-methyl-pseudouridine	LNP	BALB/c	170 µg/mL (1.4 mg/kg)	Pardi, N. et al. 2017
						BLT mice	200 µg/mL (1.4 mg/kg)	
	S057/CR57	Full-length (1.5:1)	Rabies (glycoprotein G)	Human codon optimization with GC enrichment	LNP	Swiss-Albino mice	10 µg/mL (2 mg/kg)	Thran, M.; et al. 2017
	CR8033		Influenza B (HA)				10 µg/mL (2 mg/kg)	
	Rituximab		CD20			NOD/SCID mice	N.D. (2.5 mg/kg)	
	Anti-influenza A human IgG	Full-length (N.D.)	Influenza A	N1-methyl-pseudouridine	LNP	Cynomologous NHP	4 µg/mL (0.3 mg/kg)	Sabnis, S.; et al. 2018
	CHKV-24	Full-length (N.D.)	Chikungunya virus	N1-methyl-pseudouridine	LNP	AG129 mice	14.9 µg/mL (0.5 mg/kg)	Kose, N.; et al. 2019
						Cynomologous NHP	10.1 µg/mL (0.5 mg/kg)	
							1st dose: 16.2 µg/mL (3 mg/kg) 2nd dose: 28.8 µg/mL (3 mg/kg)	
	mRNA-1944	Full-length (N.D.)	Chikungunya virus	N.D.	LNP	Human	2 µg/mL avg (0.1 mg/kg)	Zaks, T
							7.9 µg/mL avg (0.3 mg/kg)	
							10.2 µg/mL avg (0.6 mg/kg)	
							6.1 µg/mL avg (0.6 mg/kg + steroids)	
							1st dose: 7.2 µg/mL avg (0.3 mg/kg) 2nd dose: 12.9 µg/mL avg (0.3 mg/kg)	
	Trastuzumab	Full-length (2:1)	Human Her2	N.D.	LNP	C57Bl/6	57.7 µg/mL (2 mg/kg)	Rybakova, Y.; et al. 2019
**Single-chain antibodies**	VNA-BoNTA	Two VHHs fused together with albumin-binding peptide	Botulism toxin A	Human codon optimization with GC enrichment	TransIT	CD1 mice	~200–400 µg/mL (2 mg/kg)	Thran, M.; et al. 2017
	VNA-Stx2	Two VHHs fused together with albumin-binding peptide	*E. coli* shiga toxin 2				~20–50 µg/mL (2 mg/kg)	
	CD3x tumor associated antigen	RiboMAb: bispecific ScFv	Tumors expressing CLDn6, CLDn18.2 or EpCAM	N1-methyl-pseudouridine	TransIT	NSG mice	7 µg/mL (0.25 mg/kg)	Stadler, C.R.; et al. 2017
**Self-amplifying mRNA**	ZIKV-117	IRES-linked full length	Zika virus envelope protein	VEEV strain TC-83 nsP1 to nsP4 genes upstream of ZIKV117 open reading frame	NLC	C57Bl/6	1.19 µg/mL (2 mg/kg)	Erasmus, J.H.; et al. 2020
		Furin-T2A-linked full length					2.61 µg/mL (2 mg/kg)	
**Membrane bound/local delivery**	Palivizumab	Full-length (4:1) GPI anchor on heavy chain	RSV	N1-methyl-pseudouridine	None	BALB/c	N.D. (5 mg/kg)	Tiwari, P.M.; et al. 2018
	RSV aVHH	VHH with GPI anchor						
	aPGT121	Full-length (4:1) GPI anchor on heavy chain	HIV env	N1-methyl-pseudouridine	None	Sheep	210 µg/mL (2 doses of 750 µg each)	Lindsay, K.E.; et al. 2020
						Macaques	N.D. (1000 µg)	
	sPGT121	Full-length (4:1)				Sheep	80 µg/mL (2 doses of 750 µg each)	
	FcγRIV VHH-M2e	RiboBiFE; VHH bispecific Fc-receptor-engaging	Mouse FcγRIV and influenza A M2 extracellular domain	N1-methyl-pseudouridine	LNP	BALB/c	N.D. (0.25 mg/kg)	Hoecke, L.V.; et al. 2020

Abbreviations: H:L: heavy and light chain; VHH: variable heavy chain; ScFv: single-chain variable fragment; IRES: internal ribosome entry site; GPI: glycosylphosphatidylinositol; CD4bs: CD4-binding site; N.D.: not done; LNP: lipid nanoparticle; NLC: nanostructured lipid carrier; BLT: bone marrow, liver, thymus; NHP: nonhuman primate; NSG: NOD-SCID-γ.

## Data Availability

No new data were created or analyzed in this study. Data sharing is not applicable to this article.
